# Encapsulated Intracerebral Hematoma Presenting as Cerebral Abscess

**DOI:** 10.7759/cureus.15198

**Published:** 2021-05-23

**Authors:** Elise J Yoon, Jake M Jasinski, Doris Tong, Karl Kado, Boyd Richards, Robert W Mccabe

**Affiliations:** 1 Neurosurgery, Ascension Providence Hospital, Michigan State University, College of Human Medicine, Southfield, USA; 2 Radiology, Ascension Providence Hospital, Michigan State University, College of Human Medicine, Southfield, USA

**Keywords:** neurosurgery, encapsulated intracerebral hematoma, cerebral abscess, brain lesion, magnetic resonance imaging

## Abstract

Chronic encapsulated intracerebral hematoma is a rare pathology which may present after spontaneous intracerebral hemorrhage (ICH) or radiosurgery for arteriovenous malformations. A 66-year-old male presented with recent diagnosis of cerebrovascular accident (CVA) status post-treatment with tissue plasminogen activator and mechanical thrombectomy. His recent diagnoses included infective endocarditis, septic bacteremia, meningitis, and aspiration pneumonia. One month following his CVA, the patient presented with delayed altered mental status. In the setting of increasing lethargy, computed tomography and magnetic resonance imaging of the brain were performed, which suggested a brain abscess, septic emboli, and ventriculitis. The patient was taken to surgery emergently. Intraoperatively, the patient was found to have an encapsulated mass of liquid consistency. Tissue pathology demonstrated ischemic cortical tissue and hemorrhage. Multiple cultures were negative for growth. The patient was ultimately determined to have an encapsulated intracerebral hematoma. Encapsulated intracerebral hematoma should be a part of the differential diagnosis when presented with a brain abscess in the setting of a patient who is at risk of ICH.

## Introduction

Chronic encapsulated intracerebral hematoma (CEIH) is difficult to diagnose because of its tendency to mimic other brain lesions [1-4]. Patients may develop CEIH after spontaneous intracerebral hemorrhage (ICH), and in more recent years, CEIH has been reported after radiosurgery for arteriovenous malformations (AVMs) [4-11].

Brain abscesses have an estimated incidence of 1.3 in 100,000 in the United States [12]. One-third of the cases are due to hematogenous spread from cardiac, pulmonary, or dental infections. Patients may present with headaches, fever, or focal neurologic deficits [13]. While mortality associated with a brain abscess is approximately 10%, mortality related to the complication of ventriculitis is as high as 85% [13,14]. The most specific study for intracranial abscess is diffusion-weighted imaging (DWI) sequence on magnetic resonance imaging (MRI) [15]. DWI has 96% sensitivity and specificity for differentiating abscesses from other cystic intracranial lesions [16]. On MRI, brain abscesses demonstrate restricted diffusion on DWI sequence, as well as ring enhancement with contrast.

Here, we describe the case of an encapsulated intracerebral hematoma that had both imaging and clinical findings consistent with an intracerebral abscess. We sought to provide evidence that although CEIH is rare, it should be included in the differential diagnoses associated with radiographic features consistent with intracerebral abscess.

## Case presentation

Patient information

A 66-year-old male was seen at the inpatient rehabilitation unit after a cerebrovascular accident (CVA) status post-treatment with tissue plasminogen activator (tPA) and mechanical thrombectomy. The patient’s recent diagnoses included infective endocarditis, septic bacteremia, meningitis, and aspiration pneumonia. At the time of the presentation to our inpatient rehabilitation unit, he was receiving intravenous antibiotics.

Clinical findings

The patient had residual left-sided weakness from his recent CVA. He presented to our institution one week after his CVA with increasing lethargy and a waxing and waning mental status.

Timeline

**Figure 1:**
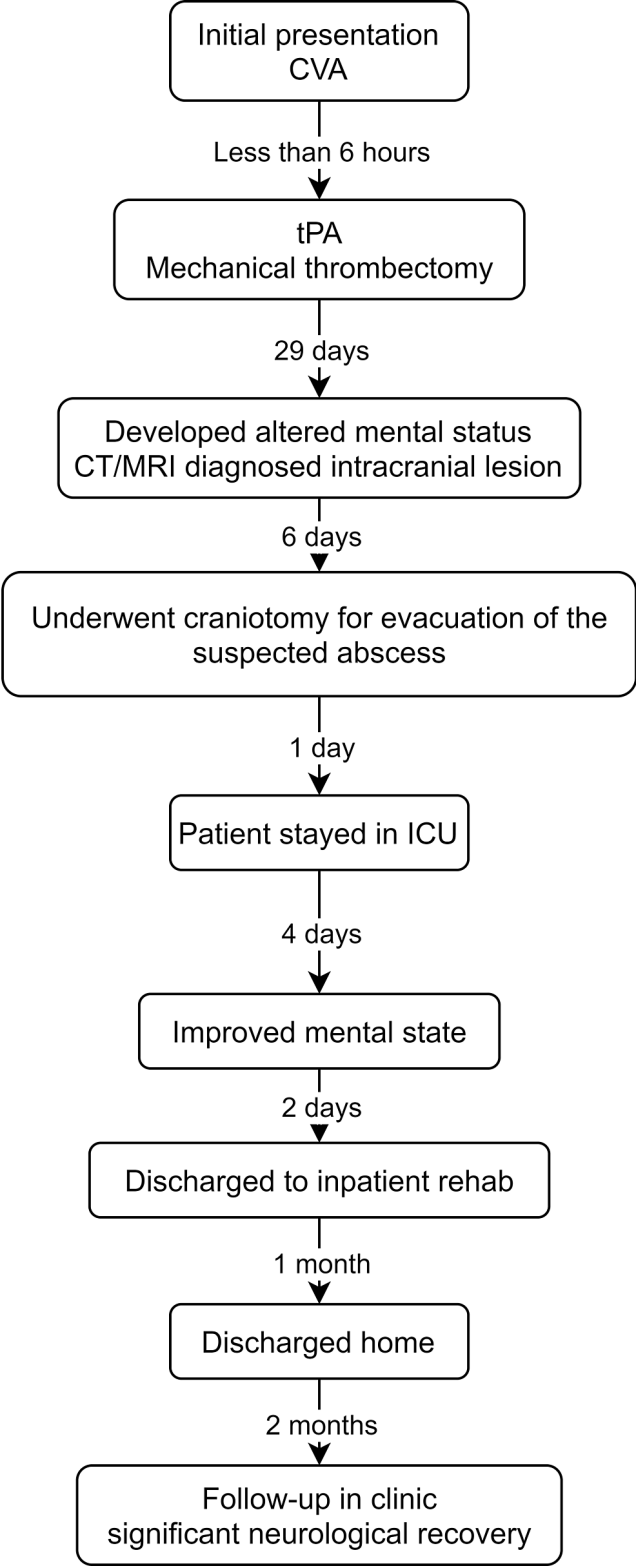
Patient presentation and treatment timeline. tPA, tissue plasminogen activator; CT, computed tomography; MRI, magnetic resonance imaging; ICU, intensive care unit

A timeline of the patient’s presentation and treatment is shown in Figure 1.

One month later, the patient was discharged home. Upon a two-month follow-up in the clinic, the patient was doing well and demonstrated significant improvement in his left upper extremity weakness.

Diagnostic assessment

**Figure 2:**
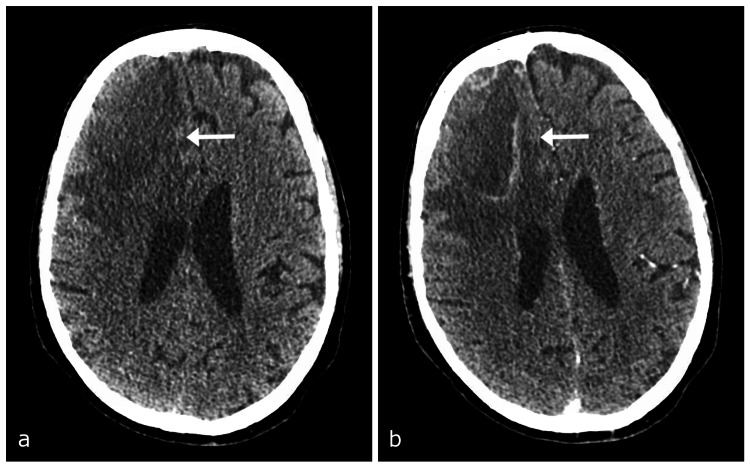
Initial imaging demonstrating a ring-enhancing lesion with surrounding vasogenic edema and mass effect in the right frontal lobe. (a) CT head without contrast and (b) with contrast. CT, computed tomography

**Figure 3:**
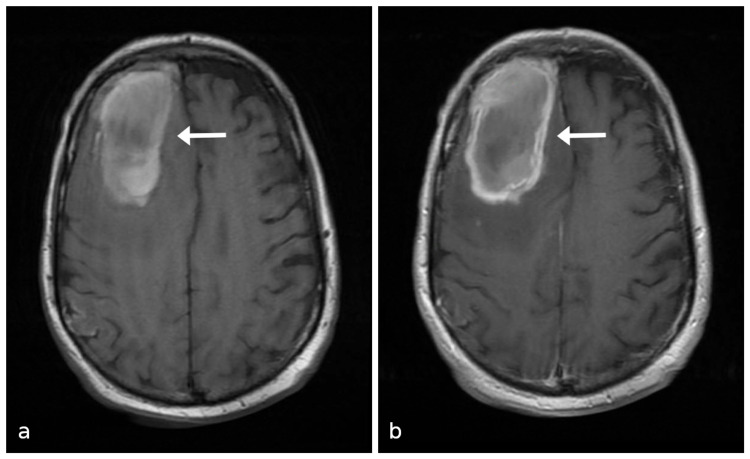
(a) MRI T1 pre-contrast and (b) post-contrast sequences demonstrating right frontal ring-enhancing lesion. MRI, magnetic resonance imaging

**Figure 4:**
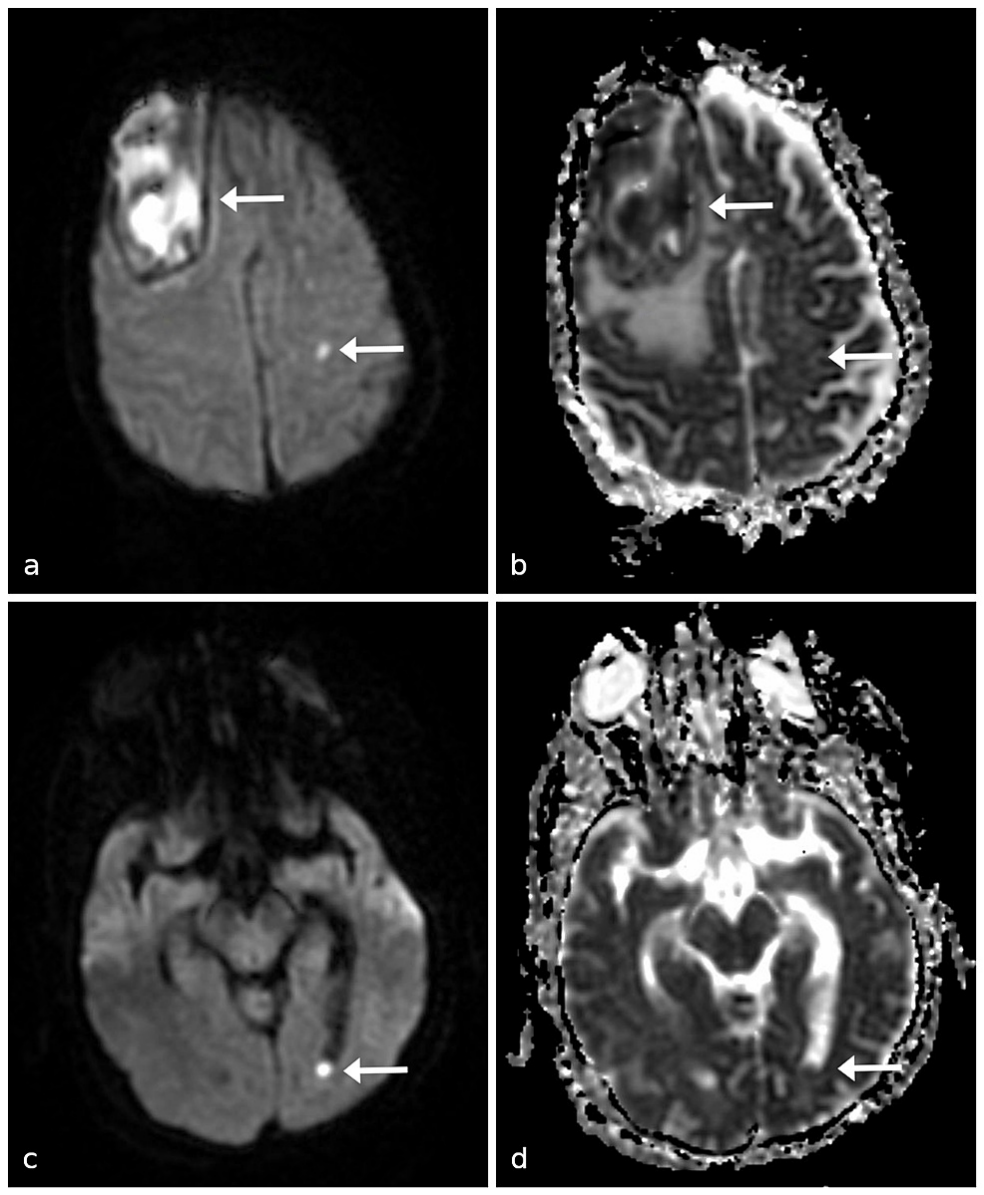
(a) MRI DWI and (b) corresponding ADC map showing restricted diffusion within the right frontal lesion; (c) MRI DWI and (d) corresponding ADC map showing restricted diffusion in the dependent lateral ventricle. MRI, magnetic resonance imaging; DWI, diffusion-weighted imaging; ADC, apparent diffusion coefficient

**Figure 5:**
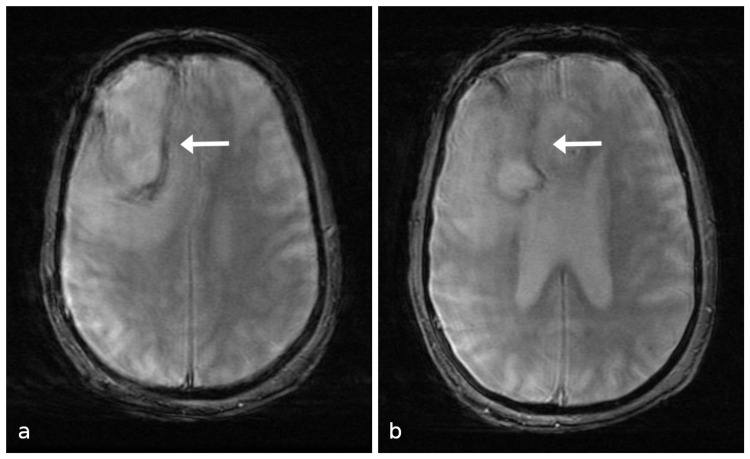
(a) and (b) MRI GRE sequences showing some susceptibility artifact in the periphery of the lesion with little to none within the lesion. MRI, magnetic resonance imaging; GRE, gradient echo

Computer tomography (CT) of the head demonstrated a 7.1-cm right frontal, intra-axial lesion with surrounding vasogenic edema, causing a significant mass effect (Figure 2). MRI demonstrated ring enhancement and areas of restricted diffusion of the lesion (Figure 3). The DWI sequence revealed scattered foci of restricted diffusion in the bilateral corona radiata suggestive of septic emboli. There was dependent debris in the lateral ventricles consistent with purulent material (Figure 4). Gradient echo sequence showed a small amount of susceptibility artifact around the primary lesion periphery with little to none within the lesion itself (Figure 5). These imaging findings were consistent with the diagnosis of cerebral abscess or rapidly proliferating malignancy.

Therapeutic intervention

**Figure 6:**
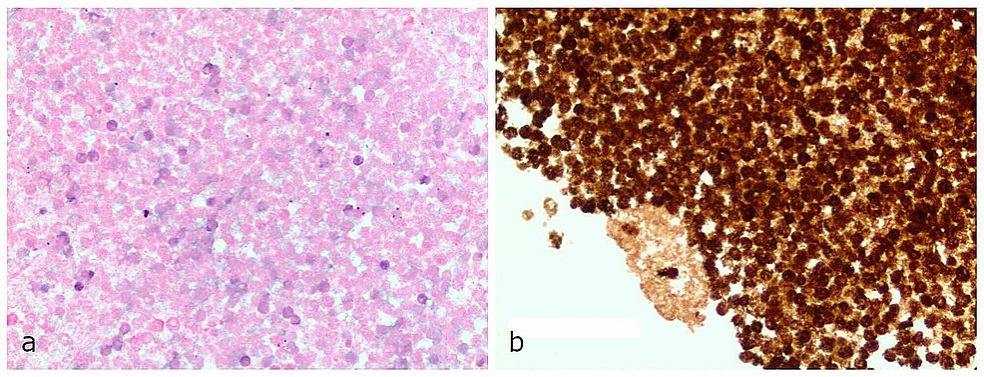
(a) H&E stain and (b) IHC stain for myeloperoxidase, both demonstrating leukocytic infiltrate. H&E, hematoxylin and eosin; IHC, immunohistochemistry

Neurosurgery was consulted due to the suspected diagnosis of cerebral abscess. The patient was taken to surgery emergently in the setting of increasing lethargy, left-sided paresis, and possible ventriculitis. A right frontal craniotomy for evacuation of the suspected abscess was performed using StealthStation™ (Medtronic, Dublin, Ireland) navigation. Intraoperatively, the lesion appeared encapsulated with a clear plane demarcating the normal brain. There was no frank purulence. The lesion was removed piecemeal. Grossly, the lesion was of liquid consistency, dark red, and with yellow-orange-tinged sections. Pathology reports of the surgical specimen identified the tissue as cerebral cortical tissue with ischemic changes and hemorrhage. The samples stained positive for myeloperoxidase, indicative of leukocytic infiltrate, which was suggestive of either an infarct or abscess (Figure 6). Gram staining of the specimen revealed no organisms, and there was no growth in cultures.

Follow-up and outcomes

Postoperatively, the patient continued to have a fluctuating mental status that slowly improved. On postoperative day six, the patient was discharged from the intensive care unit to the inpatient rehabilitation facility. One month later, the patient was discharged home. Upon a two-month follow-up in the clinic, the patient was doing well and demonstrated significant improvement in his left upper extremity weakness.

## Discussion

CEIH remains rare, and due to its variable radiographic appearance, may be mistaken for a tumor or a brain lesion [1-11]. Recent case reports have shown CEIH occurrences as a late complication of AVM radiosurgery, with 37 cases described as of 2020 [5,10]. Based on the MRI findings of the patient, the primary differential diagnosis included abscess, tumor, and hematoma. According to medical records from the outside hospital, the patient received tPA and mechanical thrombectomy for his stroke. However, due to the delayed presentation of the patient’s altered mental status, a hemorrhage or hematoma was considered to be a less likely diagnosis of his encephalopathy. The overall clinical picture, which included endocarditis, meningitis, and sepsis, suggested an intracerebral abscess.

While the DWI sequence showed restricted diffusion within the lesion, the area was not as large, and the intensity of the signal on the DWI and the corresponding apparent diffusion coefficient was not as strong as would be expected with an abscess. The restricted diffusion in the dependent lateral ventricles and scattered areas of the corona radiata suggested an embolic phenomenon, further contributing to the likelihood of an infectious process.

Cai et al. recently described CT and MRI characteristics of CEIH in a retrospective review of five patients from 2009 to 2013. The study demonstrated that on CT imaging, CEIH was visualized as quasi-circular or elliptical cystic lesions with ring enhancement similar to brain abscesses. These findings were not specific enough to make a diagnosis of CEIH. On MRI, CEIH was demonstrated as lesions with avid ring enhancement and intracapsular bleeding. The intracapsular bleeding showed varying degrees of T1 and T2 hyperintensity depending on chronicity. The study suggested that ring enhancement with intracapsular bleeding on MRI can be helpful in the diagnosis of CEIH [17].

## Conclusions

While CEIH is a rare phenomenon, it has become increasingly important to consider this pathology in the differential diagnoses to better guide patient management. In patients with a complicated clinical picture suggestive of an intracerebral abscess, a detailed patient history, radiographic assessment, and a comprehensive list of differential diagnoses will help make the diagnosis of CEIH.
